# Epigenetic Mechanisms Responsible for the Transgenerational Inheritance of Intrauterine Growth Restriction Phenotypes

**DOI:** 10.3389/fendo.2022.838737

**Published:** 2022-03-31

**Authors:** Thu Ngoc Anh Doan, Lisa K. Akison, Tina Bianco-Miotto

**Affiliations:** ^1^ School of Agriculture, Food and Wine, Waite Research Institute, University of Adelaide, Adelaide, SA, Australia; ^2^ Robinson Research Institute, University of Adelaide, Adelaide, SA, Australia; ^3^ School of Biomedical Sciences, University of Queensland, Brisbane, QLD, Australia

**Keywords:** intrauterine growth restriction, uteroplacental insufficiency, small for gestational age, transgenerational transmission, epigenetic mechanisms, cardiometabolic disease, kidney dysfunction

## Abstract

A poorly functioning placenta results in impaired exchanges of oxygen, nutrition, wastes and hormones between the mother and her fetus. This can lead to restriction of fetal growth. These growth restricted babies are at increased risk of developing chronic diseases, such as type-2 diabetes, hypertension, and kidney disease, later in life. Animal studies have shown that growth restricted phenotypes are sex-dependent and can be transmitted to subsequent generations through both the paternal and maternal lineages. Altered epigenetic mechanisms, specifically changes in DNA methylation, histone modifications, and non-coding RNAs that regulate expression of genes that are important for fetal development have been shown to be associated with the transmission pattern of growth restricted phenotypes. This review will discuss the subsequent health outcomes in the offspring after growth restriction and the transmission patterns of these diseases. Evidence of altered epigenetic mechanisms in association with fetal growth restriction will also be reviewed.

## Introduction

Intrauterine growth restriction (IUGR) refers to poor growth during pregnancy, which results in babies being born small for gestational age (SGA), and with low birth weight (LBW) (1). One of the common causes of IUGR is uteroplacental insufficiency (UPI), in which the placenta functions poorly, causing an insufficient supply of oxygen and nutrients to the developing fetus (2).

There is a high prevalence of IUGR worldwide, especially in developing countries [approximately 27% of all live births (3)], which is a significant concern, as epidemiological studies have shown that being growth restricted is associated with an increased risk of developing chronic diseases later in life (1, 4–9). In addition, various animal models have shown that IUGR offspring develop kidney dysfunction and cardiometabolic disease later in life (2, 10–15). Interestingly, these IUGR phenotypes are sex-specific and their transmission is multigenerational through both the maternal and paternal lines (11–14, 16–18).

The underlying mechanisms of how IUGR predispose offspring to chronic disease later in life remains to be determined. However, epigenetic mechanisms may be involved as they have been shown in several animal studies to be potential mechanisms for the multigenerational transmission of disease (17).

## Intrauterine Growth Restriction and Chronic Disease Risk

### Hypertension and Kidney Disease

**Figure 1:**
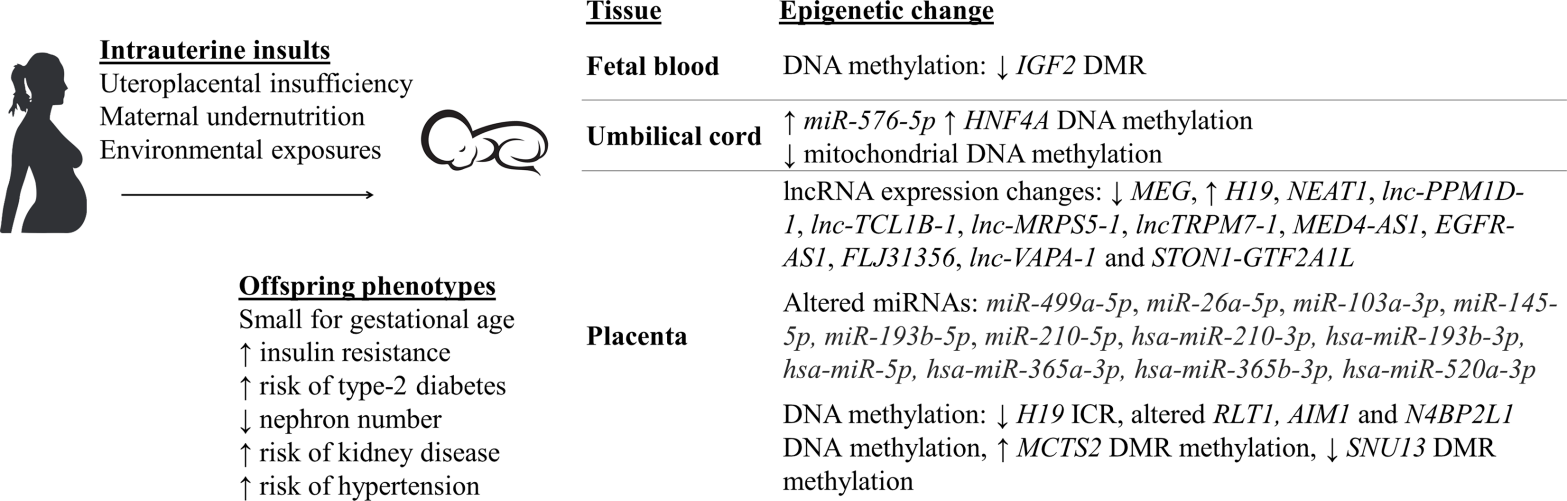
Small for gestational age (SGA) babies who were exposed to intrauterine insults have altered epigenetic mechanisms and aberrant physiological changes, predisposing them to an increased risk of developing various chronic diseases later in life (5–8, 19–40).

Epidemiological studies in humans have reported that growth restricted infants have an increased risk of developing chronic diseases later in life [**Figure 1** (5–8, 19–24)]. For instance, IUGR children at 6 years of age have been shown to have a 1.8 times higher risk of developing hypertension compared to non-IUGR children (6). Additionally, individuals born SGA had increased systolic and diastolic blood pressure by 4.5 and 3.4 mmHg, respectively, at the age of 50 (5). When these results were adjusted for confounding factors such as sex, age, and body-mass index, IUGR was still significantly associated with hypertension (5, 6). In other studies, when sex is taken into consideration, the development of hypertension in association with LBW can produce conflicting results. For example, there was one study that found an association in IUGR males only (24), while a different one found an association only with IUGR females (23). However, differences in the size of the study (15600 vs 976 children), method of measuring blood pressure (one-time systolic and diastolic blood pressure measurement vs 24h systolic blood pressure measurement), and the age of examined children [3-6 years old (24) vs 6-16 years old (23)] may be factors that contributed to the observed sex-specific differences. In line with this finding, an inverse relationship was found between birthweight and blood pressure of IUGR infants in a study that examined 1310 junior high school students (20). However, this relationship was then lost as the children reached adolescence (12-14 years of age), even when adjusted for confounding factors. This suggests that there might be a possible adaptation mechanism in the adolescents to overcome IUGR-related hypertension.

Unlike the examination of hypertension by measuring blood pressure, the precise determination of kidney disease mostly requires more invasive measurement methods, such as counting of glomerular number after organ collection and sample sectioning (7, 8). Therefore, few studies are carried out in humans, especially in growth restricted infants, to evaluate the association between IUGR and kidney disease. However, papers published by Wang et al. in 2014 (9) and 2016 (41), respectively, were two of the rare studies that investigated the effect of human aberrant fetal growth environment on kidneys of fetuses. In both studies, fetuses and their kidney samples were collected from mothers who terminate their pregnancy due to preeclampsia (9), placental abruption, deformities of fetuses, and other intrauterine insults (41). Both papers reported negative effects that IUGR had on the fetuses, including significantly low birth weight (< 2 kg), approximately 0.4 times less nephron number, increased expression of pre-apoptosis proteins within kidney tissues (9), and reduced renal renin-angiotensinogen RNA levels by half the non-growth restricted fetuses (41). This is significant, as the renin-angiotensinogen system is known to play a crucial role in maintaining the sodium homeostasis within the kidney, as well as regulating blood pressure, especially during pregnancy (41). These papers are consistent with studies that have shown a decline in glomerular number (more than 20%) in low birth weight individuals who died from cardiovascular disease as adults, in comparison to normotensive people (7, 8). Together, these studies suggest an important contribution of the kidney to hypertension development in IUGR individuals.

**Figure 2:**
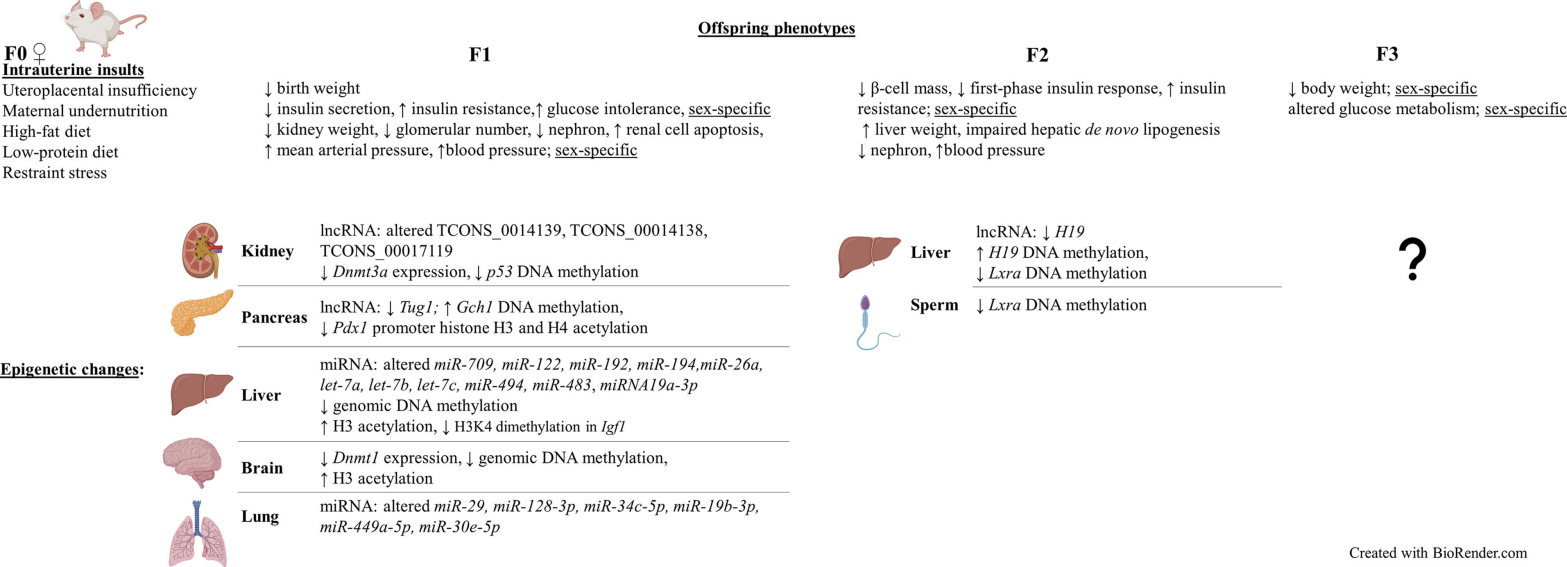
Intrauterine growth restriction (IUGR) phenotypes are sex-specific and can be transmitted to subsequent generations, including the restricted F1 and non-restricted F2 and F3 offspring (intergenerational). Similar to human studies, altered epigenetic mechanisms such as non-coding RNA modifications, DNA methylation, and histone modifications were also found in these offspring. Results obtained from differerent rat and mouse IUGR models (2, 10–15, 18, 42–70).

Using different animal models, the association between IUGR and the development of chronic diseases can also be evaluated [**Figure 2** (2, 10–15, 18, 42–57)]. In the early 2000s, the association between IUGR induced by UPI and blood pressure level was studied using a model in which placental insufficiency was established by placing silver clips around the abdominal aorta and on the branches of uterine arteries of pregnant rats at day 14 of gestation, which severely reduced blood flow between mother and the fetus (43). UPI-induced rats produced LBW offspring, 12% lighter in weight compared to control, with an increased risk of developing hypertension in both IUGR males and females, as their mean arterial pressure at 8 weeks of age was 12 mmHg higher than the control (43). However, at 12 weeks of age, only the increased mean arterial pressure in F1 male offspring was still significant, suggesting a sex-specific hypertension maintenance mechanism. There was no statistically significant association between the observed increased arterial pressure and renal function of the offspring found in this study. Glomerular filtration rate, effective renal plasma flow and 24-hour sodium excretion were not different in IUGR rats compared to the control, even when they were adjusted for kidney weight (43). Meanwhile, the bilateral uterine vessel ligation model produced restricted F1 male offspring that had higher blood pressure and an enlargement of the heart’s left ventricle at 22 weeks of age, compared to the control, as a consequence of persisting high blood pressure (10). Lower body weight and glomerular number (clusters of capillaries in the kidney, reduced by 27% of the control) were also reported at 6 months of age (10). These results were reproducible in other studies, with lower kidney weight (measured at postnatal day 1 and 7) and nephron deficit (at 18 months of age) occurring in both sexes and hypertension (at 18 months of age) being present only in male rats (2, 11, 53). Glomerular hypertrophy, an outcome to compensate for the IUGR-related glomerulus reduction, was found to be higher in the F1 growth restricted male rats compared to females at day 120 after birth, suggesting a sex-specific response of the growth restricted offspring towards kidney injury (15). Similarly, 18 month old growth restricted female rats had preserved mesenteric and renal arterial smooth muscle and endothelial function, which may in part explain why they did not develop hypertension (48). However, the mechanisms behind this remains to be identified. Interestingly, the transmission of hypertension and kidney diseases is multigenerational, as reduced nephron number, left ventricular hypertrophy and hypertension were reported in the non-restricted F2 generation (13, 14).

Apart from rats, studies of UPI-induced IUGR in other animal models [e.g. rabbit (71) and guinea pig (72)] also support the association between IUGR and reduced glomerular number and/or hypertension in the growth restricted offspring. IUGR induced by other intrauterine causes was also shown to be associated with hypertension or aberrant renal function and development (50, 52, 56). For example, F0 pregnant rats were fed a 50% deficit food intake diet throughout pregnancy to produce growth restricted F1 offspring (56). F1 males were mated with control healthy females to produce the F2 generation (paternal line). In a normoxia environment (where the oxygen concentration is normal), mean pulmonary arterial pressure, right ventricular hypertrophy index, and media wall area thickness were not significantly different between IUGR and the control males, in both generations. However, F1 and F2 male rats that were placed in an oxygen-deficient chamber for 2 weeks showed an increase in all three mentioned parameters, indicating an increased risk of developing pulmonary arterial hypertension later in life (56). In line with this finding, the expression of endothelin-1 (ET-1), a vasoconstrictor that is important for cell proliferation, cell migration, and blood vessel development, was significantly increased in pulmonary vascular endothelial cells (PVECs) extracted from F1 and F2 IUGR males. This led to aberrant PVEC proliferation, migration, and angiogenesis, all of which are signs of pulmonary vascular endothelial dysfunction (56). On the other hand, in 6-month-old LBW restricted rats whose mothers received only 50% the calories during pregnancy, there was a significant reduction in kidney weight (maximal value only reached 91% of the control) and glomerular number (by 27% the control) (52). Meanwhile, a low-protein diet (reduced by 11% of the control) in pregnant rats resulted in a significant decrease in glomerular number (by 22.6% the control; at 3 months of age) and increased renal cell apoptosis of LBW F1 offspring (50).

### Diabetes

Besides hypertension and kidney dysfunction, diabetes is another disease that has been shown to be associated with IUGR. Women whose birth weights were less than 2.5 kg [typically the clinical definition for LBW (73)] have a 1.83 times higher risk of developing type 2 diabetes as they age compared to women with birthweights above this threshold (19, 21). Decreased insulin-stimulated glucose uptake, or insulin resistance, one of the common hallmarks of type 2 diabetes, was also reported in IUGR young adults whose birth weights were below the 10^th^ percentile for their gestational age (22).

Different animal models can be used to study the association between IUGR and the development of type-2 diabetes, such as UPI model that has metabolic characteristics comparable to that of humans (18, 42, 45), IUGR rats induced by maternal calorie restriction (57, 74), or IUGR fetal sheep induced by exposing pregnant ewes to an environment with highly increased humidity and temperature (75). When both uterine arteries of pregnant rats are ligated at day 19 of gestation to imitate UPI occurring in pregnancy, F1 growth restricted rat offspring had significantly lower birth weight (5.96 g) compared to the sham control offspring (7.00 g) (42). Rat offspring in both restricted and control group then reached relatively similar body weights at approximately 7 weeks of age. However, as the IUGR F1 rats aged, they had significantly reduced insulin secretion of β-cells (by half the control at 1 week of age and completely absent at 26 weeks of age), insulin-resistance and glucose-intolerance hence hyperglycemia (42). Similar findings were also reported in other studies that applied the same UPI-inducing method of uterine arteries ligation (18, 45). Three months old growth restricted F1 rats developed hepatic insulin resistance, which was represented by its impaired insulin function in controlling the hepatic glucose production (HGP) important for maintaining blood glucose equilibrium (1.6 times higher HGP in IUGR rats compared to the control) (45). A decrease by 50% of pancreatic insulin content was also reported in the LBW growth restricted rats compared to control at the same time point of age (18). Moreover, there was a sex-specific reduction of β-cell mass in these restricted offspring compared to the control, with 40% and 50% reduction in IUGR males and females, respectively (18).

Similar to the observations for hypertension and kidney disease risks in IUGR animal studies, both the F1 and F2 generations are at a higher risk of developing diabetes, suggesting that there is a multigenerational transmission of IUGR phenotypes (12). When growth restricted F1 female rats were mated with healthy males, 6-month-old F2 offspring also had altered pancreatic β-cell mass (reduced by 29% in males and increased by two-fold the control in females) and first-phase insulin response (reduced by 35% in males and 38% in females) (12). The sex-specific differences in pancreatic β-cell mass between 3 months old F1 rats (18) and 6 months old F2 rats might be due to the difference in time point in which they were examined. For instance, at 6 months of age, female rats may have developed compensatory mechanisms for the disease. Additionally, as these defects were resolved when the rats aged [determined at 12 months of age (12)], male rats may have also developed similar mechanisms at a later age. However, this remains to be shown. In a different IUGR model where F0 pregnant rats were injected with the corticosteroid dexamethasone, from day 15 to 21 of gestation, F2 offspring had reduced birth weight and F2 5-week-old males developed glucose tolerance, represented by a significant increase in the activity of hepatic phosphoenolpyruvate carboxykinase (PEPCK), an enzyme that is involved in glucose metabolism (58). Additionally, these F2 growth restricted males were reported to have higher plasma glucose level at 4 months of age, and higher basal insulin level at 6 months of age, compared to the control (58). Similarly, when F0 pregnant rats received a restricted diet (food intake reduced by 50% the control) from day 11 to 21 of pregnancy, the effect of IUGR on insulin resistance was also seen in a multigenerational pattern (57). Specifically, F1 restricted females were also given a restricted diet from day 1 to day 21 postnatal. At 2 months of age, F1 females were mated with control males, and F2 1-day-old embryos were transferred to control recipient females. F2 female offspring from the IUGR group had significantly higher liver weight, baseline fasting plasma glucose, and insulin concentrations, despite a similar weight from birth to 15 months postnatal, compared to the control (57). The F2 IUGR group also developed insulin resistance at 15 months of age, represented by reduced plasma glucose/insulin ratio during glucose tolerant test, and lower concentration of plasma membrane-associated GLUT4, a protein that plays an important role in insulin-dependent glucose transport into skeletal muscles. Reduced function of PKCζ, an enzyme involved in insulin-signalling pathway, was also found in skeletal muscle of 15 month old F2 females in the IUGR group (57). Likewise, in a model of *in utero* low-protein consumption in rats, there was an adverse effect of IUGR on the glucose metabolism of F3 offspring at approximately 2 months of age (46). To be specific, there was a significantly higher fasting plasma glucose level in F3 females compared to sham. Meanwhile, there was a significant increase in insulin level of F3 males compared to sham at both fasting stage and 30 minutes after the glucose injection (46), suggesting that IUGR phenotypes are sex-specific and their transmission can be intergenerational. In line with this finding, reduced body weight at days 1 and 7 after birth by 0.5 g, compared to the control offspring, was also reported in the F3 rats whose grandmothers were exposed to restraint stress during pregnancy (55). Additionally, these animals were reported to have sensorimotor dysfunction at postnatal day 7, as their response time during the inclined plane test was significantly slower compared to the control (55). On the other hand, altered glucose tolerance and insulin secretion could be improved (determined in F1 male rats at 6 months of age) by cross-fostering the UPI-induced growth restricted offspring to a sham control mother for lactation, a period important for offspring development (47). This proposed that there could be reversal strategies for IUGR-related diseases and/or solution to modify their effects on the growth restricted offspring. However, intervention studies are beyond the scope of this review. Additionally, it should be noted that when IUGR was caused by a severe maternal protein-restriction diet (e.g. 5 g of protein/100 g of diet) during pregnancy, postnatal catch-up could be impaired (59). Male and female rats at 6 months of age had significantly increased fasting serum glucose level (20% and 25% the control values in F1 and F2 generation, respectively), despite being fed a control diet during lactation (59). F1 and F2 male offspring also developed insulin resistance at 6 months of age (59).

In summary, the above observations of IUGR infants having an increased risk of developing various diseases later in life are in line with the Developmental Origins of Health and Disease hypothesis, proposing that adverse events that occur during the maturation of gametes, at conception and early embryonic development can program long-term risks of chronic diseases in the LBW offspring (76). As the world-wide prevalence of type 2 diabetes, chronic kidney disease and hypertension is significantly high [6.28% for diabetes, 9.1% for kidney disease, and approximately 30% for hypertension (in adults) (77–79)], there is an urgency for researchers to investigate IUGR and its mechanisms in programming chronic diseases in humans. Nevertheless, due to the complexity and ethical rules in human research, most in-depth experiments that study IUGR are carried out in rodents. Additionally, animal models provide a mechanism for investigating the impact of IUGR across multiple generations and to determine the possible molecular mechanisms involved.

## Intrauterine Growth Restriction and the Associated Altered Epigenetic Mechanisms

### Epigenetic Mechanisms

Epigenetics can be described as heritable modifications to the chromatin that regulate gene expression without altering the DNA nucleotide sequence (80). An example of a modification that creates such changes is DNA methylation. DNA methylation involves the DNA methyltransferase-catalysed addition of a methyl group to a DNA cytosine base (81). In mammals, DNA methylation happens primarily at CpG sites, that is, a cytosine adjacent to a guanine base in the 5’-3’ direction. DNA methylation at gene regulatory regions such as promoters is associated with gene silencing (82). Additionally, DNA methylation has an important role in other processes such as X-chromosome inactivation and imprinting (83).

Another epigenetic modification is histone modifications. These include histone acetylation, which is the addition of an acetyl group to the lysine residue of the nucleosomal core histones’ N-terminal tail (81). Histone deacetylation, specifically at histones H3 and H4 is associated with gene repression (84). In addition to this, regulatory non-coding RNAs are known to be involved in transcriptional, post-transcriptional, and translational regulation hence are also involved in gene regulation (85, 86). Two large subsets of non-coding RNAs are long non-coding RNAs which are > 200 nucleotide-long, and short non-coding RNAs like microRNAs (miRNAs), short interfering RNAs (siRNAs), and piwi-interacting RNAs (piRNAs) which are all less than 200 nucleotide-long (87). Altered expression of both miRNAs and lncRNAs have been shown to be associated with altered histone modifications and DNA methylation status of genes (88, 89).

### Studies of Blood Samples

IUGR has been shown to be associated with altered epigenetic mechanisms [**Figure 1** (25–38)].

Blood samples from the Dutch Hunger Winter famine were used to investigate DNA methylation from individuals exposed to reduced calorie intake in very early (60 people) or late (62 people) gestation (90). Although there was no significant difference in birth weight among the individuals (26), there was a decrease by 5.2% in DNA methylation of the *IGF2* imprinted gene differentially methylated region (DMR) in people exposed to famine in early gestation, compared to the same-sex siblings who were not exposed to famine during pregnancy. Whilst, people exposed to famine in late gestation had no altered DNA methylation (90). This observation suggests the importance of the timing of exposure to intrauterine insults, specifically during the early developmental stage, in which epigenetic mechanisms within the fetus is programmed and may be permanently maintained into adulthood. In a different study, blood samples from 24 IUGR infants were investigated using Illumina Human Methylation 450 k array to analyse differences in genome-wide DNA methylation and gene expression, compared to data from 12 control healthy infants (60). Within the IUGR group, 5460 differentially methylated CpG loci from 2254 genes were identified. Using Kyoto Encyclopedia of Genes and Genomes database, more than 50 pathways affected by changes in the methylation status of these gene were determined, such as metabolic pathways, antigen processing and presentation, apoptosis, insulin signalling pathway, and neurological disorder pathways (60). In addition to this, increased DNA methylation, by 6.1% the control, of the type 2 diabetes-related *HNF4A* gene promoter was found in CD34+ stem cells from umbilical cord blood samples of IUGR newborns (27). More than 800 genome differentially methylated positions (DMPs) was found in leucocytes from umbilical cord blood samples of IUGR neonates, compared to the control (91). These DMPs were located within genes that are critical for key cellular processes that impact the fetal growth and development, such as organogenesis, metabolism, and immunity. *D-loop* hypomethylation of mitochondrial DNA found in fetal cord blood samples was also reported in IUGR neonates who were exposed to placental insufficiency (32). The hypomethylation was in association with higher mitochondrial biogenesis (i.e. increased mitochondrial DNA levels), which is a possible mechanism to compensate for reduced oxygen by UPI. Results in these studies were all adjusted for other complications that may have occurred during pregnancy such as gestational hypertension, gestational diabetes and preeclampsia (27, 32, 91).

Besides DNA methylation, altered expression of miRNAs have been recently reported in human umbilical cord tissues collected from IUGR pregnancies (35). To be specific, the study included samples from IUGR children with or without growth catch-up at 1 and 6 years of age, and control children who were born appropriate for gestational age (35). At 1 year of age, the expression of a miRNA *miR-576-5p*, which is known to be involved in kidney and liver diseases, was significantly enhanced in IUGR with catch-up children compared to both IUGR without catch-up and control children (35). Moreover, within the IUGR with catch-up group, *miR-576-5p* expression was shown to have a significant association with weight, height, catch-up weight, and catch-up height of the children, after being adjusted for confounding factors such as sex, gestational age, maternal smoking status, etc. Besides the mentioned parameters, at 6 years of age, *miR-576-5p* expression was also shown to be associated with renal fat, suggesting an important role of *miR-576-5p* in cardiometabolic diseases, and that alterations of this miRNA due to IUGR may increase the risk of developing these diseases later in life (35).

### Studies of Kidney Tissues

Animal studies that specifically focused on altered epigenetic mechanisms in the UPI-induced IUGR offspring have also been carried out in different organs and tissues [**Figure 2** (44, 49, 51, 53, 54, 61–68)]. Decreased expression, by 19% the control, of *Dnmt3a*, a gene that is responsible for *de novo* DNA methylation was found in kidney tissues of F1 IUGR rats at embryonic day 20 (53). Meanwhile, decreased apoptosis-suppressing *Bcl-2* gene expression and increased pro-apoptotic protein-encoding *Bax* and *p53* expression were identified in kidneys of F1 IUGR rats at term, which was associated with reduced glomerular number (by 23% the control) of rat pups (44). Correlatively, there was reduced DNA methylation of CpG islands at the promoter region (by 56.3% the control) of *p53* (44).

Whilst, significantly altered expression of three long non-coding RNAs (lncRNAs) (TCONS_0014139, TCONS_00014138, and TCONS_00017119) at day 1 and day 10 postpartum (pn1 and pn10), confirmed by both microarray and qPCR, were found in kidneys of LBW male rats whose mothers were also fed a low-protein diet during pregnancy (51). The altered expression of these lncRNAs were associated with altered mRNA expression at pn1 and pn10 of *MAPK4*, which encodes for a protein that involves in renal ureteric bud morphogenesis. Additionally, the aberrant expression of these lncRNAs is also correlated with a decrease in nephron number of LBW rats at pn1, suggesting an important role of them in nephron endowment (51). Furthermore, altered expression of *Cdkn1c* and *Kcnq1*, two imprinted genes that are regulated by the *Kcnq1ot1* lncRNA, was found in kidney tissues of UPI-induced IUGR rats at day 1 after birth (53). However, further research is required to investigate whether changes to *Kcnq1ot1* was the epigenetic mechanism that affected the imprinted gene expression in this study.

### Studies of Liver Tissues and Pancreas Tissues

Similar to results obtained from blood samples and kidney tissues, abnormal DNA methylation have also been found in hepatic tissues from IUGR studies (49, 65, 66). Importantly, in hepatic tissues, the multigenerational transmission and reversibility of the altered epigenetics was detected in F2 non-restricted offspring (66). Growth restricted F1 rats that underwent intrauterine UPI were fed with either a control diet or essential nutrient supplemented (ENS) diet (i.e. rich in methyl donors) and bred spontaneously to produce the F2 offspring (66). Within the F2 generation, 21-day-old rats whose F1 mothers received a control diet had statistically reduced DNA methylation of the *H19* gene promoter (7% less than the sham lineage), in association with reduced *H19* expression (0.4-fold the sham lineage) (66). Meanwhile, 21-day-old F2 rats whose F1 mothers received ENS diet had increased *H19* promoter methylation (34% more than the sham lineage), with a 6.6-fold increase in *H19* expression (66). In line with this finding, F2 offspring of pregnant mice that were fed with only 50% the control group’s food intake had significantly lowered expression of the *Lxra* gene (p < 0.01) in their liver tissues, which plays a key role in *de novo* lipogenesis (49). Hepatic *de novo* lipogenesis was also impaired in the F2 adult mice (49). Furthermore, this was associated with statistically reduced methylation within the 5’UTR region of *Lxra*, both in the sperm samples of IUGR F1 males and liver samples of non-restricted F2 fetuses and adult mice. Therefore, it is suggested that there was a multigenerational transmission of altered *Lxra* methylation within both F1 and F2 generations (49). Meanwhile, one of the first studies to investigate whole-genome DNA methylation from pancreatic islet samples in the UPI-induced IUGR 7-week-old male rats discovered 1912 differentially methylated loci compared to the control, most of which occurred within the non-coding intergenic sequences between genes rather than promoter regions (65). Interestingly, the differential methylation was 45kb upstream of genes known to be important for homeostasis-maintaining processes (e.g. *Fgrf1*, *Gch1*, and *Vgf*) and were correlated with altered expression of these genes (65).

UPI-induced IUGR in several animal studies has also been shown to be associated with altered histone modifications (61–63, 69, 92). Histone H3 hyperacetylation, increased to 233% the control value, was detected in the liver of UPI-induced IUGR newborn rats, in association with hepatic genomic DNA hypomethylation (reduced methylation by 13.7% the control at day 21 after birth) (61). On the other hand, significantly reduced dimethylation at H3K4 in the *Igf1* region was reported in livers of IUGR rats whose mothers had a food restriction during pregnancy (69). Meanwhile, locus-specific assessment of the *Pdx1* gene, a gene important for β-cell development and function, showed loss of *Pdx1* promoter H3 and H4 acetylation at 6 months of age and significant DNA hypermethylation (increased by 51.3% the control) in the pancreatic islets of F1 IUGR adult rats, and was associated with silencing of *Pdx1* (mRNA level reduced by 50.4%) (63). This may contribute to the later onset of type-2 diabetes in the growth restricted offspring.

In regards to non-coding RNAs, not many IUGR studies have been carried out to investigate their changes in the growth restricted offspring. Nonetheless, in agreement with results obtained from the placentas in human studies, reduced expression of the lncRNA *H19* and reduced DNA methylation status of its promoter region were reported in hepatic tissues of F2 growth restricted rats whose grandmothers (F0) underwent UPI (66). In hepatic tissues of F1 growth restricted mice whose mothers were fed with a high-fat diet pre-, during and post-pregnancy, there was also a significant reduction in expression of the miRNAs, including *miR-709, miR-122, miR-192, miR-194, miR-26a, let-7a, let-7b, let-7c, miR-494* and *miR-483* (64). Interestingly, a major of the altered miRNAs are predicted to have a common target, which is methyl-CpG binding protein 2 (64). In a different study where F0 pregnant mice were fed a low-protein/calorie-deficit (-40%) diet from week 3 of gestation, and growth restricted pups were cross-fostered to 3 different groups right after birth, either normal milk feeding (6 pups/dam), overfed (3 pups/dam), or nutrition restriction (10 pups/dam), significantly reduced H3K4me3 (trimethylated histone H3 on lysine 4) region at the *Akt1* gene, a gene that is known to play an important role in insulin resistance, was found in livers of 3-month-old IUGR males that either received normal milk feeding or were overfed, in association with reduced expression of *Akt1* (93). Interestingly, higher protein level of PTEN, one of the *Akt* activation inhibitors, was also found in livers of overfed 3-month-old males. In addition to this, significantly decreased levels of circulating *miRNA19a-3p*, a miRNA that acts to regulate PTEN, were found in both normally fed and overfed IUGR males (93). These finding hence suggests an association between altered epigenetic mechanisms and the risk of developing insulin resistance later in life of IUGR offspring. Indeed, compared to F1 healthy control males, males that were either under nutrition restriction or overfed both had an increase in sensitivity to insulin at 3 months of age (93). At 12 months of age, the sensitivity to insulin increased for the nutrition restriction group but attenuated for the overfed group. Meanwhile, IUGR males that received normal milk feeding showed no difference in insulin sensitivity compared to healthy control males at 3 months of age. However, at 12 months of age, they developed insulin resistance (93). On the other hand, in pancreatic islets of growth restricted mice whose mothers were fed a low-protein diet, the expression of *Tug1*, a lncRNA that involves in diabetes and tumour development, were significantly lower at 1 day, 8 weeks, and 12 weeks post-partum, compared to the control (54). The aberrant glucose tolerance observed at 10 weeks old IUGR mice could be partially rescued by injection of 150 µg of *Tug1* overexpression sequence, suggesting that *Tug1* may play an important role in the mouse pancreatic development and function (54).

### Studies of Placental Tissues

Altered DNA methylation of genes that are important for fetal growth and development has been reported in placentas from both human and animal IUGR pregnancies (34, 39, 70, 94). For example, placenta samples from healthy and complicated human pregnancies were investigated using the Illumina Infinium Human Methylation450 BeadChip arrays (HM450k) array platform (67 samples) and quantitative pyrosequencing (127 samples) (39). Specifically, 35 DMRs that are expressed across tissues (ubiquitous) were identified. In general, DNA methylation status of all DMRs was not significantly different between complicated pregnancies and the control group. However, DNA hypermethylation was found at the *MCTS2* DMR, while hypomethylation was found at the *SNU13* and *H19* ICR in IUGR placentas (39). Additionally, RT-PCR and Sanger sequencing confirmed that *H19* hypomethylation results in the biallelic expression of *H19* in the IUGR group. Similarly, a loss of methylation in *SNU13* is associated with increased expression of this gene in the IUGR placentas. Interestingly, despite a similar DNA methylation status compared to that of the control, there was an increase in expression of *ZNF331* and a decrease in expression of *PEG10* and *ZDBF2* in the IUGR placentas (39). For DMRs that are placenta-specific, the same HM450k array data was used, and results were also confirmed using pyrosequencing. Out of 32 placenta-specific DMRs, methylation status of *AIM1* and *N4BP2L1* was significantly different in the IUGR group compared to the control. However, using microfluidic-based quantitative RT-PCR analysis, only four placenta-specific genes that had altered expression in IUGR samples compared to the control were identified, all of which were reduced in IUGR, including *ADAM23*, *GPR1-AS1, LIN28B*, and *ZHX3* (39). In line with this, altered DNA methylation of CpG island 1 of *RLT1*, a gene known to be important in placental development, was found in placenta samples from SGA and severe SGA foetuses, compared to healthy controls (34). Whilst, genome-wide DNA methylation patterns were investigated in placentas of IUGR identical twins who shared the same placenta (monochorionic twines) and had significant growth difference, represented by birthweight variations in the range of 21-59% (94). In placental tissues of IUGR twins, altered DNA methylation status (with differences larger than 10% compared to healthy control twins) were identified in DMRs that overlapped the promoters of 8 genes that are known to be important for lipid metabolism and neural development, including *DECR1, ZNF300, DNAJA4, CCL28, LEPR, HSPA1A/L, GSTO1*, and *GNE* (94). These results were still significant after being adjusted for twins’ sex, gestational age, and maternal age. Interestingly, *DECR1* and *GSTO1*, the two genes play a role in fetal growth, have also been altered in other IUGR studies in animal and human singleton pregnancies, suggesting potential shared molecular mechanisms in comparison to the IUGR growth-discordant monochorionic twins (94). Meanwhile, at embryonic day 10.5, altered DNA methylation was found within 20 different DMRs of imprinted loci from the placentas of F2 mice, whose grandmothers had a hypomorphic mutation in methionine synthase reductase (*Mtrr*), a gene that is important for methyl group utilisation and maternal folate metabolism (70). Changes in DNA methylation status of these DMRs were associated with changes in expression of imprinted genes such as *Zdbf2*, *Igf2*, and *Dlk1*, all of which play a key role in fetal development (70). As expected, growth restriction, delayed development, and defects of different organs including brain, heart and placenta were also found in these offspring (70), suggesting a multigenerational transmission of IUGR phenotypes and altered epigenetic mechanisms.

Altered expression of long non-coding RNAs that are important for angiogenesis, inflammation fetal growth has also been reported in placentas collected from pregnancies that are affected by IUGR (25, 28, 31, 38, 40). The investigation of 30 IUGR and 46 gestational age-matched non-IUGR placentas revealed decreased expression of *MEG3*, a lncRNA that is involved in placental and fetal growth, by more than 50% in the IUGR samples compared to the non-IUGR control (25). In line with this, the expression of *H19*, another lncRNA that is important for fetal development, was also reduced by half the non-IUGR control in IUGR placentas (31). This reduction in *H19* expression was shown to be strongly correlated with a 50% decrease of expression of the type III TGF-β receptor (TβR3), one of the downstream signalling molecules that control trophoblast cell migration and invasion (31). However, in a different study, the expression of *H19* was shown to be similar between IUGR and non-IUGR placental tissues (28). Nonetheless, in IUGR placentas, there was a significantly lower DNA methylation status in the imprinting control region 1 (ICR1), which regulates the expression of *H19*, in comparison to the control (28). Increased expression of another lncRNA *NEAT1*, a gene expression regulator which expression in usually up-regulated in human cancers, was also seen in placentas from IUGR pregnancies with a 4.14-fold increase in IUGR placentas compared to the control (29). In contrasts, in a different study, *NEAT1* expression in the placentas was not statistically different between IUGR and the non-IUGR group (95). Differences in sample size, ethnicity, or maternal age might be an explanation for the differences in *H19* and *NEAT1* expression in IUGR placentas in the above studies. In a recent study, altered expression of 133 lncRNAs (36 increased in expression and 98 decreased in expression, in comparison to non-IUGR control) was reported in placentas from 12 pregnant women whose pregnancies were complicated with IUGR (38). Interestingly, the overexpression of several lncRNAs such as *lnc-PPM1D-1*, *lnc-TCL1B-1*, *lnc-MRPS5-1*, *lncTRPM7-1*, *MED4-AS1*, *EGFR-AS1*, *FLJ31356*, *lnc-VAPA-1* and *STON1-GTF2A1L* in the IUGR group was also found in placentas from the pregnancy group affected by preeclampsia (38). Most of these lncRNAs have been shown to play a role in pathways that lead to placental ischemia, which results in reduced blood supply to the placenta (38). This suggests that these pregnancy complications might act *via* some shared mechanisms and/or there are similar signalling pathways can be activated by them.

Similar to the observations for lncRNAs, there are miRNAs that have been shown to be altered in placentas from both IUGR and preeclampsia pregnancies, such as *miR-499a-5p*, *miR-26a-5p*, *miR-103a-3p*, *miR-145-5p* (30), *miR-193b-5p* (36), *miR-210-5p* (37), *hsa-miR-210-3p, hsa-miR-193b-3p, hsa-miR-5p, hsa-miR-365a-3p, hsa-miR-365b-3p*, and *hsa-miR-520a-3p* (33), most of which play a role in cellular functions, including cellular differentiation, migration and invasion, suggesting shared signalling pathways and mechanisms between these pregnancy complications.

### Studies of Other Tissues

The focus of this review is on the risk of developing renal and cardiometabolic diseases, such as hypertension and diabetes, in association with IUGR induced by UPI. Therefore, most of the studies reported are on either blood samples, kidneys, livers, pancreas, or placentas. However, it should be noted that there are other tissues that can also be affected by IUGR, such as lungs or brains. For example, when F0 pregnant rats were fed a 50% deficit food intake diet throughout pregnancy, in PVECs extracted from the F1 and F2 IUGR rats, there was a significant enrichment of H3K4me3 regions in F1 IUGR males, and a significant reduction in DNA methylation at ET-1 CpG sites in both F1 and F2 IUGR males, compared to the control (56). Interestingly, ET-1 CpG methylation was also significantly reduced in F1 IUGR rat sperm, suggesting epigenetic modifications as a potential mechanism for the multigenerational transmission of these IUGR phenotypes, *via* the paternal line (56). In line with this finding, altered expression of various miRNAs were found in lung tissues at day 10, day 21, and 5 months after birth of IUGR rat offspring whose mothers were either undernourished (68) or fed with a low-protein diet (67) during pregnancy. Most of these miRNAs (*miR-29, miR-128-3p, miR-34c-5p, miR-19b-3p, miR-449a-5p*, and *miR-30e-5p*) are involved in lung development and injury-repair (67, 68). Microarray analysis and homologous analysis of brain tissues containing hippocampus from growth restricted F1 rats whose mothers received a 50% reduction in food intake throughout pregnancy also revealed 49 rat genes that are homologous in humans, and had a negative correlation between gene expression and DNA methylation status (60). Most of these genes are involved in metabolism pathways, nervous system dysfunction, cancer, and immune response regulation (60). Increased cerebral total H3 acetylation (to 157% of control values), decreased genome-wide DNA methylation (to 52.8% the control), and decreased CpG island methylation (to 65.0% the control) were also found in brains of IUGR rat offspring (62). Simultaneously, the expression of cerebral chromatin-affecting enzymes DNA methyltransferase 1 and methyl-CpG binding protein 2 were decreased in neonatal IUGR rats, with the mRNA abundances of 50% and 38% the control values, respectively (62).

## Discussion

From the above evidence it is clear that the effects of intrauterine growth restriction may have on the long-term health and well-being of infants are extensive. Diabetes, hypertension, and kidney dysfunction in growth restricted offspring are the most common diseases that were shown to be related to IUGR. Moreover, as the frequency of this pregnancy complication and its associated diseases is high, especially in developing countries, there is a need to determine the mechanisms of how aberrant phenotypes are programmed and transferred to subsequent generations. In comparison to humans, the examination of tissues and organs in animals is more accessible for scientists, ethically. Therefore, the proposed potential mechanisms for the diseases’ multigenerational transmission will come from in-depth studies of animal experimental models. Additionally, sex-specific expression of the diseases’ phenotypic outcomes was also observed in animal offspring. Therefore, further assessments are required to determine whether epigenetic mechanisms are responsible for the sex-specific differences of IUGR related diseases. Subsequently, future studies may focus on investigating similar mechanisms and markers in humans, which will help identify people who are at risk and/or identify potential prevention strategies for these diseases.

## Author Contributions

TD performed the literature search, interpreted the data, and wrote the manuscript. TB-M critically revised the manuscript and supervised the project. LA critically revised the manuscript. All authors contributed to the article and approved the submitted version.

## Conflict of Interest

The authors declare that the research was conducted in the absence of any commercial or financial relationships that could be construed as a potential conflict of interest.

## Publisher’s Note

All claims expressed in this article are solely those of the authors and do not necessarily represent those of their affiliated organizations, or those of the publisher, the editors and the reviewers. Any product that may be evaluated in this article, or claim that may be made by its manufacturer, is not guaranteed or endorsed by the publisher.
